# Lessons from the frontline: Documenting the pandemic emergency care experience from the Pacific region – Infrastructure and equipment

**DOI:** 10.1016/j.lanwpc.2022.100516

**Published:** 2022-07-06

**Authors:** Megan Cox, Deepak Sharma, Georgina Phillips, Rob Mitchell, Lisa-Maree Herron, Claire E. Brolan, Gerard O'Reilly, Sarah Körver, Mangu Kendino, Penisimani Poloniati, Berlin Kafoa

**Affiliations:** a Faculty of Medicine and Health, The University of Sydney, Sydney, Australia; b The Sutherland Hospital, Sydney, NSW, Australia; c NSW Ambulance, Sydney, Australia; d Colonial War Memorial Hospital, Suva, Fiji; e School of Public Health and Preventive Medicine, Monash University, Melbourne, Australia; f Emergency Department, St Vincent's Hospital Melbourne, Melbourne, Australia; g Emergency & Trauma Centre, Alfred Health, Melbourne, Australia; h School of Public Health, Faculty of Medicine, The University of Queensland, Brisbane, Australia; i Centre for Policy Futures, Faculty of Humanities and Social Sciences, The University of Queensland, Brisbane, Australia; j Port Moresby General Hospital, Papua New Guinea; k Emergency Department, Vaiola Hospital, Nuku'alofa, The Kingdom of Tonga; l Public Health Division, Secretariat of the Pacific Community, Suva, Fiji

**Keywords:** COVID-19, Emergency care, Pacific, Health infrastructure, Health equipment, PPE, Donations, Ventilators, Oxygen, Building blocks, PICTs

## Abstract

**Background:**

The COVID-19 pandemic highlighted challenges for all health systems worldwide. This research aimed to explore the impact of COVID-19 across the Pacific especially with regards to emergency care (EC) and clinicians’ preparations and responses.

**Methods:**

A collaboration of Australia and Pacific researchers conducted prospective qualitative research over 18 months of the pandemic. In this three phase study data were gathered from Emergency Clinicians and stakeholders through online support forums, in-depth interviews and focus groups. A phenomenological methodological approach was employed to explore the lived experience of participants. This paper discusses the findings of the study regarding the EC building block of ‘Infrastructure and Equipment.’

**Findings:**

Pre-existing infrastructure and equipment were not sufficient to help control the pandemic. Adequate space and correct equipment were essential needs for Pacific Island emergency clinicians, with donations, procurement and local ingenuity required for suitable, sustainable supplies and facilities. Adequate personal protective equipment (PPE) conferred a sense of security and increased Health Care Workers willingness to attend to patients.

**Interpretation:**

Investing in adequate infrastructure and appropriate equipment is crucial for an effective response to the COVID-19 pandemic. The sustainability of such investments in the Pacific context is paramount for ongoing EC and preparation for future surge responses and disasters.

**Funding:**

Phases 1 and 2A of this study were part of an Epidemic Ethics/World Health Organization (WHO) initiative, supported by Foreign, Commonwealth and Development Office/Wellcome Grant 214711/Z/18/Z. Co-funding for this research was received from the Australasian College for Emergency Medicine Foundation via an International Development Fund Grant.

**Research in context**

**Evidence before this study**

Large scale disasters place huge stresses on all countries’ health infrastructure and resources. The recent historic event of COVID-19 has highlighted worldwide competition and pressure for health-related equipment despite preparedness of countries and regions. The relevance of these issues to emergency care (EC) delivery during the pandemic has not been explored, especially in the context of the Pacific region.

**Added value of this study**

This prospective, qualitative study investigated the experiences of Pacific EC clinicians responding to the pandemic and examined issues regarding infrastructure and equipment of most concern to EC stakeholders in Pacific Island Countries and Territories. We found that EC facilities were overburdened and unprepared for this pandemic and HCWs (Health Care Workers) were extremely vulnerable in this inequity. We highlight the resourcefulness and resilience of EC HCWs in implementing solutions relating to health care space and supplies despite enormous challenges.

**Implications of all the available evidence**

The study emphasised the importance of well-resourced health system to the delivery of safe and timely EC during the pandemic, just as under routine conditions. Health Infrastructure requires prepared facilities with surge capacities and flexible spaces directed by HCWs who are knowledgeable and empowered. Personal protective equipment (PPE) has been shown to be a key driver for HCW safety and must be available, appropriate and accountable. Inequity of health equipment distribution is unfair, unethical and has worldwide ramifications, as shown by the continued inequity in access to COVID vaccines. Equipment purchases and donations should be tailored to local needs, with local clinician partnerships focusing on suitability, safety, sustainability and integration into existing health systems.

Alt-text: Unlabelled box

## Introduction

There is abundant literature advocating for EC in Low- and middle-income countries (LMICs) and demonstrating it is pragmatic, inexpensive and highly cost effective.^1^^,^^2^^,^^3^ In 2019, the 72nd World Health Organization (WHO) Assembly adopted a resolution confirming the importance of Trauma and Emergency care (EC) as part of a health system and emphasising the need for EC in establishing universal health coverage.^4^ At that time WHO Director General Dr Tedros Adhanom Ghebreyesus said “We have simple, affordable and proven interventions that save lives. All people around the world should have access to the timely, life-saving care they deserve”. However, less than one year later, the COVID-19 pandemic highlighted significant health system weaknesses with disruptions in countries of all income levels worldwide.^5^

LMICs across the Pacific region continue to be impacted by the COVID-19 pandemic, with access to every form of health care reduced as a direct result.^6^ The burden differs due to varying border closures and other policies, but the region is suffering economic burden, food insecurity, and lack of access to safe water and essential health care.^6^ Some island nations have had no COVID 19, some small outbreaks, and others major ongoing burdens.^7^ From early on, Pacific EC leaders have been on the frontline of health care responses with responsibilities extending from triage and clinical management of patients with COVID-19 to health system leadership and coordination.^8^

**Figure 1:**
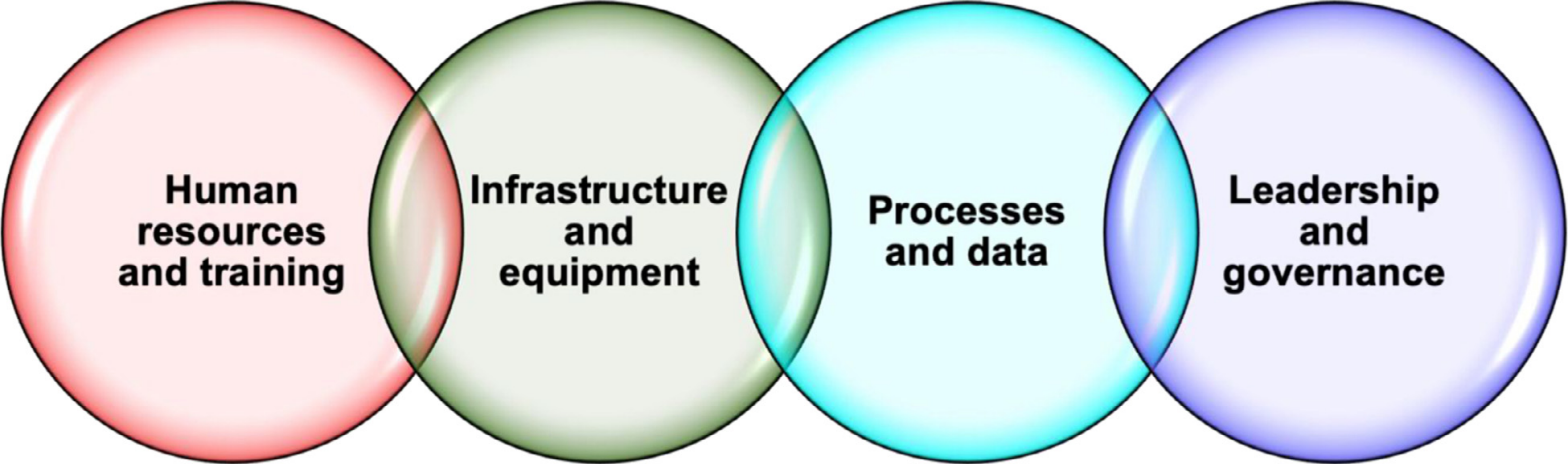
WHO health system building blocks, adapted for the Pacific EC context and this qualitative research project.

This rapid, collaborative, qualitative research project started in 2020 as an exploration of the experiences of EC clinicians in Pacific LMICs early in the COVID-19 pandemic. Qualitative data obtained from online regional EC support forums, key informant interviews and focus groups has been analysed in thematic approaches including using key themes of the WHO health system Building Blocks (BB)s adapted for the Pacific EC context (Figure 1).^9^^,^^10^

This BB framework for strengthening health systems was established in 2007 to promote a common understanding of health systems, how to strengthen them and identify health delivery gaps.^9^ Infrastructure and Equipment issues were specified in two of these BBs, highlighting that a good health service delivers effective, safe, quality health interventions with equitable access to essential medical products, vaccines and technologies of assured quality, safety, efficacy and cost-effectiveness.^9^ Pacific EC health care workers (HCWs) encountered the pandemic at differing stages of health system readiness but common themes are found and explored. This paper describes the key operational themes, enablers and barriers involving the BB infrastructure and equipment from this project. We highlight lessons learnt and hope this paper supports Pacific HCWs and colleagues worldwide as we navigate this pandemic together.

## Methods

### Study design

The study methods are described in detail elsewhere.^11^ In brief, this study was conducted as a collaboration between Australian and Pacific Island Country and Territory (PICT) researchers, and employed prospective, qualitative research methods grounded in a phenomenological methodological approach.^12^^,^^13^

Data were collected from EC clinicians and other relevant stakeholders across PICTs in three phases between March 2020 and July 2021 (Table 1). Informed consent was obtained from research participants through a mixture of written and verbal consent. Semi-structured interview and discussion guides developed by the research team were used in Phases 2 and 3.

**Table 1:** Data collection phases and participants.

Phase 1	Online support forums via ZOOM	• 13 online support forums hosted by SPC and ACEM, between March and October 2020 • > 80 active participants (EC clinicians and stakeholders) from PICTs (and some non-Pacific countries) voluntarily engaged in online discussion
Phase 2	In-depth interviews via ZOOM	• Semi-structured interviews (45-90 minutes) with 13 key informants in Fiji, Kiribati, Palau, Papua New Guinea, Samoa, Solomon Islands, Timor Leste, Tonga and Vanuatu • Purposively selected: key informants coordinated EC in a PICT during the COVID-19 pandemic
Phase 3	Focus group discussions via ZOOM	• Three focus groups, with EC stakeholders from Pacific regions of Micronesia (Federated States of Micronesia, Kiribati, Marshall Islands, Nauru, Palau, the northern Pacific states), Polynesia (Cook Islands, Samoa, Tokelau, Tonga, Tuvalu, other Small Island states) and Melanesia (Fiji, Papua New Guinea, Solomon Islands and Vanuatu, as well as Timor Leste)

Data collected at each phase were digitally recorded with participant permission, transcribed verbatim, and subsequently de-identified to protect participants’ anonymity. All data were preliminary coded using QSR NVivo^14^ using a hybrid inductive (data driven)^15^ and deductive^16^ approach. Deductive codes were derived from the WHO health system building blocks adapted for the Pacific EC context (Figure 1).^10^ The subset of data related to Infrastructure and Equipment was then thematically analysed by MC and DS. Emerging themes and tentative findings were presented to the broader research team at several online meetings for verification through discussion and data triangulation.

Ethics approval was provided by The University of Sydney Human Research Ethics Committee (Reference 2020/480) and registered with Monash University Human Research Ethics Committee (Reference 28325). Research protocols for Phases 1 and 2A of the research were also reviewed by the World Health Organization's AdHoc COVID-19 Research Ethics Review Committee (Protocol ID CERC.0077) and declared exempt. Reporting of study data adheres to Enhancing the Quality and Transparency of Health Research (EQUATOR)^17^ and Standards for Reporting Qualitative Research (SRQR)^18^ guidelines.

## Results

Four key themes related to infrastructure and equipment were identified from our analysis of data presented by EC HCWs in the Pacific. These were explored in depth and summarised also in Table 2 with relevant sub themes listed with enablers and barriers.

**Table 2:** Themes, Enablers and Barriers for Infrastructure and Equipment.

Enablers	Barriers
Theme 1: Mismatched health care facilities to community needs	
Planning and communication “*When the country did the planning for a lot of things that were planned for were planned to stay, even after is gone then we can still continue to use these things.”* *“… the Director at that time ... brought me this list and so I had to go through that list and tick what we needed. There were ventilators, PPEs on that list. So, we got everything that I asked for so I must say that was pretty good.”*	Inadequate space “*As we speak there is overcrowding outside our ED now and that's one of the challenges we try to control.”* *“I think at this point in time with the number of sick patients that we are seeing our space is very limited so it might compromise our staff safety.”* *“But, the clinicians, the nurses, everybody on the ground were just voicing that, you know: ‘We can't deal with it, we are not trained to deal with this, our facilities won't be able to deal with this, what do we do?’”*
EM staff experience in disaster *“The emergency staff have really taken the forefront of planning, to the extent that they've actually argued with national health ministers, which is great.”*	No surge capacity; Lack of pandemic or disaster plans *“What would happen if our unit is full, where are we going to keep the patients? The hospital team is also planning to have a separate area in our hospital in case the number increases. The number is continually increasing every day.”*
Theme 2: Lack of access and availability to PPE	
Effective advocacy and leadership *“I think that the process initially may have been a bit slow to start, but now it's running quite well and there's a process to get more into the country, and also a system to get it into the hospital facility, and also another process to keep enough in supply within our emergency department.”* *“And staff, meaning from the doctors right down to the cleaners, they all come under you. And they have families whom you have to consider, so their safety is of paramount importance. Even the clerks too. So yeah, that's how I see it, you kind of take care of everybody who's working in your department.”* *“Initially it wasn't that easy. You had to pursue, keep on pushing, ‘cause it wasn't in constant supply. You had to talk and talk and talk and talk until, like, right now, up to date, I'm kind of settled now, because we have been channelled through the disease control where all the stocks come in, and so now we are getting supplies constantly. But at the start it wasn't like that, I had to go here, there, everywhere, barge into somebody's office and say ‘I want this’. And, eventually, he said okay I put you with this group of people so you can easily get your PPEs.”*	Inequitable access to PPE, no local supply, closed borders “*We had issues with PPEs, we had to order from overseas as we don't have local companies here that can provide or make them.”* *“The difficulties, as I think everyone has discussed, is our PPE supplies. We've ordered quite a lot from different places. Because we've closed the borders, we cannot get those supplies in.”* *“The other issue that's come in given that all our borders and everything were closed was our supplies of other drugs that we needed – the antibiotics, some of our drugs needed in ICUs – these have been forwarded from the hospital taskforces to our national taskforce so that we can procure them – but that was the other issue, given that our borders were closed.”*
Stock control and communication, planning, organisation *‘Then our hospital itself has devised, through the infection control team, they have the usage rates, they calculate it so they can project how much more PPEs we'll need, and they're in a constant process of ordering it so that we do not run short.’* *“but now with a proper inventory of masks and PPEs we are able to regulate the usage and also to see the usages, to see you know how many is left in stock and how many we need to order.”*	Staff fears and safety concerns “*Yes, we did have that doubt, whether we had enough, or whether we'll be able to access and get an equal supply.”* *“Our staff were feeling unsafe, especially our nursing staff. They protested not to come to work. They actually had a protest to the government to provide PPEs or they weren't going to go to work. It was chaotic during this time of lockdown.”* Poorly suited PPE *“The only thing was, they gave these full body suits, you know these white full body suits, which were a little bit hot. You sweat inside them. And had only one size. We had some really big people and really small people, so they wouldn't fit in those equipment”.*
Theme 3: Competition for clinical equipment	
Re-use and recycling of equipment *“For, one of the things is that goggles and face masks, buying them from other places has been very expensive. We are now making our own face masks.”* *“Oh yes we are reusing our eye shields and goggles. They get soaked in Milton Solution for 15 mins then washed, dried and reused. …. OT has sewn their masks and also we had planned to use materials to sew masks, gowns for training purposes. And we do reuse our goggles.”*	Lack of normal critical care equipment *“In terms of intensive care, I don't think we'll be able to do that here.”* *“Up ‘til now I haven't received any equipment; it's just like minor diagnostic equipment like pulse oximeter. I just managed to get one oxygen concentrator but otherwise apart from that, nothing. So, like we're still using our ED things now so, there's nothing specifically for -COVID-19.”*
Local experts guiding international donations *“You know, we had so many funding, we had so many people coming in to want to assist …. So, every now and then, ‘Oh, what about this country? No one is mentioning it’. So, any other additional support we could get we'd draw this countries and put them in.”*	Lack of sustainability *“’Hey, we've run out of all the consumables to do with these ventilators that they sent in…. We've run out of it already. What do we do? We need some more.’ So now that goes and sits in the corner.”*
Positive equipment changes *“COVID has really improved our service in terms of identifying weak areas, or potential areas that can crack, like the nebuliser, which is, like in our ED we have the nebuliser that asthmatics come and help themselves.”*	Inadequate staff skill/ training *“The mismatch came we got the four ventilators and then realised that we don't have enough staff to run those ventilators.”* *“…even though we received those ventilators machine, we needed to have training. I don't think most of those people here especially the whole country especially the doctors including those working in referral would be able to use that ventilator.”*
Theme 4: Dealing with Donations	
Donation leading to system improvements *‘Before not many equipment or ambulance, now we have quite a number of ambulances in our hospital and ED now have equipment like oxy log. So that's something we never had in the last 20 years.”*	Mismatched needs *“We've actually had many embassies donate, which is great. We're getting equipment, but the main concern really is, basically human resources and training capacity.”*

### Theme 1: Healthcare facilities mismatched to community needs

Hospital facilities were frequently described as inadequate, poorly maintained or completely absent by participants. HCWs discussed that even with no COVID-19 in their setting, their facilities were regularly overburdened and crowded, and worried about how and where they would deal with an increased workload. These factors promoted indecision about where suspected patients would be triaged, tested and treated.

*“Our facilities were not ready, in terms of preparation … we've decided that we will handle our patients somewhere outside of the hospital.” “At the moment we have yet to establish where exactly to manage our patients. ... our hospital is under major construction, redevelopment, right now.”*

Participants mentioned that watching the COVID-19 pandemic cause rapid changes in High Income countries (HIC) led them to despair for their own communities. The speed of the pandemic's impact on equipment needs new protocols and patient volumes caused alarm for many.

*“My colleagues and I as well, were fearful, because we tend to compare ourselves to other developed countries like New Zealand and Australia, where the infrastructure and equipment is advanced, more advanced than we are. So, we tend to compare ourselves to that, and then that makes us feel – what do you call it – vulnerable and prone.”*

Once COVID-19 entered some of these Pacific Island states, EC HCWs anxiously discussed the escalating needs, rapid changes and the lack of surge capabilities of their systems.

“*What would happen if our unit is full, where are we going to keep the patients? The hospital team is also planning to have a separate area in our hospital in case the number increases. The number is continually increasing every day.”*

Many facilities had never had isolation rooms, negative pressure rooms or an intensive care unit (ICU) and the emphasis of these requirements in HICs generated much discussion.

*“We only have one working ventilator in ICU and as you know, it's a very small ICU, that's where the general population goes in. So, just to put it bluntly – no, we have not yet decided where to manage these patients.”*

### Theme 2: Lack of access and availability to Personal Protective Equipment (PPE)

Access to PPE was a major discussion point from the very first webinar with participants commenting on the usual difficulties obtaining PPE pre pandemic:

*“The hospital normally just runs out of gloves and face masks; this is something that happens all the time.”*

*“All of our clinical equipment, our masks, everything, are re-used, they always have been. So, we clean them down with soap and water and then alcohol and re-use them. Because we wouldn't have enough stock.”*

Participants complained of decreased ability to order PPE as borders closed, with many HICs stockpiling supplies and prices rising due to worldwide shortages.

*“Initially, because there was just everybody going for the same thing, you had to sort of wait in line for you to get your orders and get it out. And then there's the long wait again to find a flight, to get it out to the countries.”*

*“We do have major issue with low PPE supplies and due to closed borders. We have ordered PPE and some donations are on the way. However, we are still trying to get them into country by boat, to schedule cargo boats as flights are still closed.”*

Frequent discussions occurred between participants regarding home-made masks and face shields, how to safely recycle items while trying to control storage and supply of specific items. For example:

*“They have prepared some reusable PPEs, the gowns and the hats. They get together, they sew it and they prepare themselves. And of course, they collect monies from donations and they got these washing machines and they use to wash them.”*

Once PPE arrived, there were supply and stock control issues requiring careful, communicative leadership and open communication*.*

*“There's a lot to explore where either the staff are very comfortable to use PPE … or how the PPE is controlled within the institution or the organisation. Because when there's no PPE people get scared. When there's PPE available, then people get scared to wear it.”*

After establishing some supplies, concerns emerged about how these consumables would be replaced given most borders remained closed.

*“... we are still okay with PPEs as per the latest update from our pharmacy. But it is a lot of consumption of our PPEs, because every arrival of a plane, the number of ground handlers and porters at the airport, in addition to our health team. So right now, we should also be ordering more to keep topping up our supplies with respect to PPEs.”*

### Theme 3: Competition for clinical equipment

As countries around the world stocked up on PPE, other integral specific clinical equipment such as COVID-19 testing kits, oxygen and ventilators were also in high demand. Most participants' countries had no onsite capacity for testing and early on in many countries testing samples required transport to HICs.

*“So, there's plans for testing capacity here. We are working on getting a PCR machine. The machine has yet to arrive.”*

This led to confusion and delays as to whether or not COVID-19 was actually in the country.

*“Let's talk about the lab, you know the lab testing. That took quite a while to put them into place. And all these things, it caused unnecessary anxiety among people. We say, okay we got it here, but you don't have it until you have it in country and it's working. Or some people may have it in country and no one knows how to use the machine; there's no expertise there, or there's no whatever, the supporting stuff that you use for all these different equipment.”*

Lack of access to oxygen was particularly frustrating for many participants, with the majority normally relying on bottle supplies to be brought to facilities.

*“We only have one oxygen supplier, in the city, they supply bottled oxygen for so long. I don't whether it is a political thing or something but we haven't been able to install our oxygen plant in any of the major hospitals.”*

*“Oxygen supply was one of the major issues… we have bought 100 cylinders and we ran through 30 in the first week.“*

Participants expressed particular concern about oxygen availability in remote facilities:

“*In the remote clinics, the outer islands, the places that have nothing, really all they have for respiratory patients is their nebuliser that's not run-on oxygen…. I'm just hopeful that these oxygen concentrators will magically appear. And I guess that there's a big need for them in those more remote places that don't really have anything else at the moment.”*

Critical care facilities are uncommon in most Pacific settings, and many participants discussed that their facilities had never had an Intensive Care Unit (ICU) or negative pressure rooms.

*“In terms of intensive care, I don't think we'll be able to do that here. We don't have ventilators; we don't have a lot of stuff here. So, we're basically going to provide that level of care just from oxygenation, stuff like that.”*

Many HCWs discussed whether they would use ICU equipment for the first time in a pandemic. As one explained:

*“We have no ventilators at the moment. We have ordered some but we, most of us, don't think it's the time to start using them now, at such a critical time.”*

### Theme 4: Dealing with donations

With worldwide reduced supplies of PPE and clinical equipment, some participants reported that their countries' local partners rapidly donated supplies and other practical resources to HCWs. Participants acknowledged that countries with previous partnerships benefitted early with equipment*: “Having I think a good relationship with the donor partners as well that has helped in getting this stuff in early.”* Other countries required more direct advocacy, for example:

*“There were countries that donors would specifically come out and would want to help. And the responses from these countries were in line with the help they were getting …. So, there was different levels of support to different countries, and how countries responded I think was based mostly on the support they were able to get.”*

Participants commented that when selected donor countries reached out offering equipment it needed to be sustainable, and this was best guided by expert local input.

“*I think a big lesson here is not to get sucked up into what people are offering, what donors are offering, don't get sucked up into it. But you need to know your health systems very well. You need to know, and be confident about, what I can do and what is appropriate, now, or in the future, and work along that*” “*We need brave people like that who are able to say that, at this point of time, that is a waste of resources – yes, maybe down the line then we can go and get those – but let's put these basic systems into place and then we'll deal with that down the line.”*

However, many participants acknowledged the pandemic benefitted them with access to better equipment.

“*One of the good things for us in (my country), COVID makes a lot of things easier in terms of getting equipment. For instance, like blood gas, I don't think we would ever get this if not for COVID. So, this is something that, we are now also getting Oxylog, this is something that we would never get if it is not for COVID.”*

## Discussion and lessons learnt

This is the first study exploring infrastructure and equipment issues at the frontline of the EC COVID-19 response in the Pacific region to our knowledge. Our research aimed to capture the rich and diverse voices of EC clinicians in PICTs and document lessons learned in the pandemic. We found that despite mismatched health facilities, pandemic unpreparedness and inadequate equipment, EC HCWs across the Pacific displayed incredible resourcefulness and resilience. They planned and prepared new spaces, recycled and modified PPE and equipment, and advocated for appropriate and sustainable space and supplies. As we analysed the themes, we recognised key enablers and barriers involved in these themes and have included these in Table 2 with key supportive quotes from participants.

These themes, enablers and barriers are not new and have been discussed and highlighted many times worldwide.^1^^,^^4^^,^^9^^,^^19^ Pre-pandemic, a PICT consensus paper highlighted issues of limited space and care delivery in overcrowded and unsafe ECs in the region.^10^ COVID-19 has magnified the importance of infrastructure and equipment, as seen in the WHO's pandemic action plans that emphasised the need for designated clinical services and facility level planning to deliver essential and routine EC in COVID-19 response efforts.^20^ The infrastructure theme of mismatched facilities centred around lack of space and ED overcrowding, a known major adverse patient and staff safety issue, and well researched internationally.^21^

Despite mismatched EC Infrastructure in PICTs, HCWs involved in our research reported promoting and supervising surge responses such as triage tents and sporting facilities for COVID-19 patient care. They led facility responses improving patient flow through ECs, with reduction in access block, improved inter- hospital and inter facility communications. Their responses highlight PICT HCWs as engaged and resilient leaders able to build and support surge capacity despite limited structural resources. These skills of HCWs should be recognised and utilised to support locally led and sustainable solutions for ED Infrastructure in PICTS, alongside the consensus regional infrastructure recommendations.^10^ Recent reports show that increasing healthcare infrastructure leads to positive proactive and reactive HCW responses, even specifically in COVID-19.^22^

EC HCWs are essential to management of COVID-19 but are amongst the most vulnerable to occupational exposure.^23^ Previous studies based on the Ebola experience highlighted the conflict HCWs felt maintaining normal care when PPE was unavailable.^24^ Early in the pandemic, HCW occupational acquisition of COVID-19 and deaths resulted in multiple access to PPE campaigns on social media.^25^^,^^26^ Pacific Island nurses shared these fears and concerns, with some attempting to wear the same PPE all shift and even organising industrial action.^27^^,^^28^ HCWs with prior medical conditions are known to be more at risk of COVID-19 and these are especially prevalent in PICTs.^27^ Recommendations for increased infection prevention and control (IPC) procedures and redeployment of at risk HCWs were made^23^, but are unsuited to PICTs due to pre-existing issues of inadequate supplies, border closures, and complicated by simultaneous disasters such as dengue outbreaks and tropical cyclones.^29^

The global shortage of PPE led to exorbitant price rises and reduced bargaining power for LMICs despite international organisations advocating for equitable access.^25^^,^^30^ Lack of PPE was a major discussion point in the forums, with participants encouraging HCW advocacy and safety while looking for local solutions. Local embassies and companies donated PPE and HCWs shared experiences of making and reusing masks and face shields. A collaborative and ingenious resource, the Pacific HCW safety guide was written by forum participants out of concern for protecting HCW families and communities from COVID-19 occupational exposure.^31^ Reports of cleaning and reusing N95 masks in HICs^32^^,^^33^ inspired ingenious ideas on sustainability and re-using of consumables that would otherwise be thrown out after a single use. An oxygen resource with instructions on safely sterilising oxygen tubing and masks^33^ was widely shared. PICT HCWs frequently clean and re-use ‘single use’ items, so the concept of cleaning and reusing is not new; in fact this may be a creative environmental solution requiring further research in view of the increasing environmental disaster of health waste.^34^

There is much evidence that providing basic EC equipment to LMICs is not expensive, complex or inappropriate^35^^,^^36^ and impacts significantly in multiple disease processes.^1^^,^^2^ However, essential EC equipment gaps in LMICs still remain, recently documented in PICTs.^10^ Initially in the COVID-19 pandemic, many governments and international donors focussed on distributing ventilators in LMICs, disregarding evidence that many LMICs had no prior experience of emergency or critical care, and well known gaps in essential EC equipment, human resources and training.^37^^,^^38^ Pre-pandemic it was estimated that nine out of ten LMICs lacked pulse oximetry and access to any oxygen,^39^ which has obvious and significant logistics and safety concerns with ventilators. Discussions captured in our research revealed stark ethical, safety and logistical dilemmas PICT EC staff encountered when ventilators were offered to places who had never or infrequently used them.

**Box 1 Lessons learnt regarding Infrastructure and Equipment**

• Infrastructure needs to be increased, and built as well-designed flexible spaces
• Health Infrastructure needs to have surge capacity to respond to different emergencies
• PPE is a key driver for staff safety – must be available, appropriate and accountable
• Equipment purchases and donations should be tailored to local needs, with local clinician partnerships

Alt-text: Unlabelled box

Participants' discussions also revealed the familiarity they had with their EC equipment and how much they valued supply chain management systems, ensuring sustainable ordering and proper training and repair. Maintenance of equipment with local technical and clinical staff was highlighted, otherwise equipment risked lying idle, a well-known problem globally.^40^ A recent WHO report concluded the majority of donated ICU equipment in one region was left unopened and unused due to lack of trained staff, or unused once broken through lack of trained biomedical staff for repairs.^41^ Long term sustainable and impactful change can occur from regional EC leaders advocating for EC equipment for many types of critically unwell patients, not just COVID-19 related.^36^^,^^39^ However discussions highlighted that equipment purchased or donated without consideration of biomedical, maintenance, distribution, and economic systems, does not lead to appropriate clinical, logistic, ethical or sustainable solutions. Donations must be connected to health priorities and leaders on the ground, linked to an understanding of the system with its strengths and limitations, then tailored and supported for maximal utilisation and sustainability.^10^^,^^37^

### Limitations

Our research was conducted over the first 18 months of the COVID-19 pandemic, now changed significantly with new variants dominant (Delta) and emerging (Omicron) and vaccination issues paramount. At the time of our research, these issues were not discussed, so further research involving these would greatly benefit PICTs. Our discussions involved mostly urban hospital-based EC staff, so prehospital and remote and regional issues were not highlighted, a major consideration in an area with vast island territories. All our research and discussions were in English, but a number of the resources shared were able to be translated into Pacific languages.^31^ We look forward to mentoring and supporting further research in Pacific languages to enrich these themes.

This research was qualitative, descriptive and we encourage its interpretation alongside ‘on the ground’ research and evaluation of health infrastructure and equipment. Frequent equipment and infrastructure changes occurring worldwide and locally during the pandemic may have impacted frontline HCWs perspectives at different times. The priorities and needs of frontline EC clinicians evolved as knowledge and pandemic experience increased over time. Although we captured the equipment and infrastructure experience of Pacific HCWs early in the pandemic, the key themes identified are likely to have meaning through all stages of this and future surge events.

Furthermore, communication issues between frontline and management staff may have also impacted knowledge and interpretation of supplies and systems. For example, supplies of PPE may have been abundant in some contexts, but not readily made visible or available to frontline HCWs through fragmented clinical governance. Improving health infrastructure and equipment alone has limited effect if introduced into a weak health system with no feedback loops. The building block framework has high utility for health systems research, but can lead to simplistic interpretations of complex health systems if each block is not understood as intersecting with all others.^42^

### Summary and conclusion

The COVID-19 pandemic has demonstrated that simple, routine and affordable interventions such as PPE, oxygen and access to safe healthcare spaces are inaccessible and/or unavailable for many HCWs and patients worldwide. Just as previous pandemics such as Ebola have exposed failings in healthcare systems, COVID-19 has highlighted the fragility of health system responses, and worldwide inequity in health care. However, as previous pandemics have brought about positive changes such as vaccines and community led responses, it is hoped COVID-19 will lead to long lasting collaborative health system responses, assisted by engaged communities, resilient leaders and using many of the innovative and sustainable solutions shown by EC HCWs in PICTs in this pandemic response.

## Contributors

L.H. performed and M.C. and D.S. contributed to the thematic analysis, secondary coding and synthesis. M.C. and D.S. researched and developed the first draft of this manuscript. G.P., R.M., L.H., C.B., G.R. and S.K. provided review and approval of the final version. M.K., P.P. and B.K. provided regional perspective. All authors approved the final version.

## Data sharing statement

Study protocols and de-identified data that underpin these findings may be available (between 9 and 24 months after publication) to investigators whose proposed use of these data has been approved by an independent review committee, and subject to any restrictions imposed by relevant ethics committees, funders, the Australasian College for Emergency Medicine or the Pacific Community. Proposals to use the data may be submitted, and data made available, without investigator support.

## Declaration of interests

M.C., G.P., R.M. and G.O.R. declare they are recipients of International Development Fund Grants from the Australasian College for Emergency Medicine Foundation. G.P. reports past research funding from the Pacific Community (SPC) and visiting Faculty status at the University of Papua New Guinea and Fiji National University. Additionally, R.M. reports grants from the Australian Government Department of Foreign Affairs and Trade as well as scholarships from the National Health and Medical Research Council (NHMRC) and Monash University. G.O.R. reports that he is the recipient of a NHMRC Early Career Research Fellowship. C.E.B. reports past research consultancy funding from SPC.

## Appendix Supplementary materials

Image, application 1
