# Supplementary material for: Lessons from the frontline: Documenting the pandemic emergency care experience from the Pacific region – Infrastructure and equipment
Source: Lancet Reg Health West Pac. 2022 Jul 6;25:100516. doi: 10.1016/j.lanwpc.2022.100516 (PMC9259041; doi:10.1016/j.lanwpc.2022.100516)
Supplement: Supplementary file 1 [file mmc1.docx]

**SUPPLEMENTARY TABLES and BOX for Infrastructure and Equipment article**

**TABLES**

**Table 1. Data collection phases and participants**

| Phase 1 | Online support forums  via ZOOM | - 13 online support forums hosted by SPC and ACEM, between March and October 2020 - > 80 active participants (EC clinicians and stakeholders) from PICTs (and some non-Pacific countries) voluntarily engaged in online discussion |
| --- | --- | --- |
| Phase 2 | In-depth interviews  via ZOOM | - Semi-structured interviews (45-90 minutes) with 13 key informants in Fiji, Kiribati, Palau, Papua New Guinea, Samoa, Solomon Islands, Timor Leste, Tonga and Vanuatu - Purposively selected: key informants coordinated EC in a PICT during the COVID-19 pandemic |
| Phase 3 | Focus group discussions  via ZOOM | - Three focus groups, with EC stakeholders from Pacific regions of Micronesia (Federated States of Micronesia, Kiribati, Marshall Islands, Nauru, Palau, the northern Pacific states), Polynesia (Cook Islands, Samoa, Tokelau, Tonga, Tuvalu, other Small Island states) and Melanesia (Fiji, Papua New Guinea, Solomon Islands and Vanuatu, as well as Timor Leste) |

**Table 2. Themes, Enablers and Barriers for Infrastructure and Equipment.**

| **ENABLERS** | **BARRIERS** |
| --- | --- |
| **Theme 1: Mismatched health care facilities to community needs** | |
| **Planning and communication**  “*When the country did the planning for a lot of things that were planned for were planned to stay, even after is gone then we can still continue to use these things.”*  *“… the Director at that time ... brought me this list and so I had to go through that list and tick what we needed. There were ventilators, PPEs on that list. So, we got everything that I asked for so I must say that was pretty good.”* | **Inadequate space**  “*As we speak there is overcrowding outside our ED now and that’s one of the challenges we try to control.”*  *“I think at this point in time with the number of sick patients that we are seeing our space is very limited so it might compromise our staff safety.”*  *“But, the clinicians, the nurses, everybody on the ground were just voicing that, you know: ‘We can’t deal with it, we are not trained to deal with this, our facilities won’t be able to deal with this, what do we do?’”* |
| **EM staff experience in disaster**  *“The emergency staff have really taken the forefront of planning, to the extent that they’ve actually argued with national health ministers, which is great.”* | **No surge capacity; Lack of pandemic or disaster plans**  *“What would happen if our unit is full, where are we going to keep the patients? The hospital team is also planning to have a separate area in our hospital in case the number increases. The number is continually increasing every day.”* |
| **Theme 2:** **Lack of access and availability to PPE** | |
| **Effective advocacy and leadership**  *“I think that the process initially may have been a bit slow to start, but now it’s running quite well and there’s a process to get more into the country, and also a system to get it into the hospital facility, and also another process to keep enough in supply within our emergency department.”*  *“And staff, meaning from the doctors right down to the cleaners, they all come under you. And they have families whom you have to consider, so their safety is of paramount importance. Even the clerks too. So yeah, that’s how I see it, you kind of take care of everybody who’s working in your department.”*  *“Initially it wasn’t that easy. You had to pursue, keep on pushing, ‘cause it wasn’t in constant supply. You had to talk and talk and talk and talk until, like, right now, up to date, I’m kind of settled now, because we have been channelled through the disease control where all the stocks come in, and so now we are getting supplies constantly. But at the start it wasn’t like that, I had to go here, there, everywhere, barge into somebody’s office and say ‘I want this’. And, eventually, he said okay I put you with this group of people so you can easily get your PPEs.”* | **Inequitable access to PPE, no local supply, closed borders**  “*We had issues with PPEs, we had to order from overseas as we don’t have local companies here that can provide or make them.”*  *“The difficulties, as I think everyone has discussed, is our PPE supplies. We’ve ordered quite a lot from different places. Because we’ve closed the borders, we cannot get those supplies in.”*  *“The other issue that’s come in given that all our borders and everything were closed was our supplies of other drugs that we needed – the antibiotics, some of our drugs needed in ICUs – these have been forwarded from the hospital taskforces to our national taskforce so that we can procure them – but that was the other issue, given that our borders were closed.”* |
| **Stock control and communication, planning, organisation**  *‘Then our hospital itself has devised, through the infection control team, they have the usage rates, they calculate it so they can project how much more PPEs we’ll need, and they’re in a constant process of ordering it so that we do not run short.’*  *“but now with a proper inventory of masks and PPEs we are able to regulate the usage and also to see the usages, to see you know how many is left in stock and how many we need to order.”* | **Staff fears and safety concerns**  “*Yes, we did have that doubt, whether we had enough, or whether we’ll be able to access and get an equal supply.”*  *“Our staff were feeling unsafe, especially our nursing staff. They protested not to come to work. They actually had a protest to the government to provide PPEs or they weren’t going to go to work. It was chaotic during this time of lockdown.”*  **Poorly suited PPE**  *“The only thing was, they gave these full body suits, you know these white full body suits, which were a little bit hot. You sweat inside them. And had only one size. We had some really big people and really small people, so they wouldn’t fit in those equipment”.* |
| **Theme 3: Competition for clinical equipment** | |
| **Re-use and recycling of equipment**  *“For, one of the things is that goggles and face masks, buying them from other places has been very expensive. We are now making our own face masks.”*  *“Oh yes we are reusing our eye shields and goggles. They get soaked in Milton Solution for 15 mins then washed, dried and reused. …. OT has sewn their masks and also we had planned to use materials to sew masks, gowns for training purposes. And we do reuse our goggles.”* | **Lack of normal critical care equipment**  *“In terms of intensive care, I don’t think we’ll be able to do that here.”*  *“Up ‘til now I haven’t received any equipment; it’s just like minor diagnostic equipment like pulse oximeter. I just managed to get one oxygen concentrator but otherwise apart from that, nothing. So, like we’re still using our ED things now so, there’s nothing specifically for -COVID-19.”* |
| **Local experts guiding international donations**  *“You know, we had so many funding, we had so many people coming in to want to assist …. So, every now and then, ‘Oh, what about this country? No one is mentioning it’. So, any other additional support we could get we’d draw this countries and put them in.”* | **Lack of sustainability**  *“’Hey, we’ve run out of all the consumables to do with these ventilators that they sent in…. We’ve run out of it already. What do we do? We need some more.’ So now that goes and sits in the corner.”* |
| **Positive equipment changes**  *“COVID has really improved our service in terms of identifying weak areas, or potential areas that can crack, like the nebuliser, which is, like in our ED we have the nebuliser that asthmatics come and help themselves.”* | **Inadequate staff skill/ training**  *“The mismatch came we got the four ventilators and then realised that we don’t have enough staff to run those ventilators.”*  *“…even though we received those ventilators machine, we needed to have training. I don’t think most of those people here especially the whole country especially the doctors including those working in referral would be able to use that ventilator.”* |
| **Theme 4: Dealing with Donations** | |
| **Donation leading to system improvements**  *‘Before not many equipment or ambulance, now we have quite a number of ambulances in our hospital and ED now have equipment like oxy log. So that’s something we never had in the last 20 years.”* | **Mismatched needs**  *“We’ve actually had many embassies donate, which is great. We’re getting equipment, but the main concern really is, basically human resources and training capacity.”* |

**Box 1. Lessons learnt regarding Infrastructure and Equipment**

- Infrastructure needs to be increased, and built as well-designed flexible spaces
- Health Infrastructure needs to have surge capacity to respond to different emergencies
- PPE is a key driver for staff safety – must be available, appropriate and accountable
- Equipment purchases and donations should be tailored to local needs, with local clinician partnerships
